# High-quality crystals of human haematopoietic prostaglandin D synthase with novel inhibitors

**DOI:** 10.1107/S1744309110020828

**Published:** 2010-06-24

**Authors:** Sachiko Takahashi, Toshiharu Tsurumura, Kosuke Aritake, Naoki Furubayashi, Masaru Sato, Mari Yamanaka, Erika Hirota, Satoshi Sano, Tomoyuki Kobayashi, Tetsuo Tanaka, Koji Inaka, Hiroaki Tanaka, Yoshihiro Urade

**Affiliations:** a Confocal Science Inc., Japan; b Osaka Bioscience Institute, Japan; c Maruwa Foods and Biosciences Inc., Japan; d Japan Aerospace Exploration Agency, Japan

**Keywords:** human haematopoietic prostaglandin D synthase, prostaglandin D_2_, allergy, inflammatory reaction, microgravity

## Abstract

Human haematopoietic prostaglandin D synthase (H-PGDS; EC 5.3.99.2) produces prostaglandin D_2_, an allergic and inflammatory mediator, in mast cells and Th2 cells. H-PGDS has been crystallized with novel inhibitors with half-maximal inhibitory concentrations (IC_50_) in the low nanomolar range by the counter-diffusion method onboard the Russian Service Module on the International Space Station. The X-ray diffraction of a microgravity-grown crystal of H-PGDS complexed with an inhibitor with an IC_50_ value of 50 n*M* extended to 1.1 Å resolution at 100 K using SPring-8 synchrotron radiation, which is one of the highest resolutions obtained to date for this protein.

## Abstract 2

High-quality crystals of human haematopoietic prostaglandin D synthase in complex with novel inhibitors were obtained in microgravity.

## 1. Introduction

Prostaglandin (PG) D_2_ is a lipid mediator related to immunity and inflammation through the activation of two types of receptors, the D-­type prostanoid receptor (DP) and the chemoattractant receptor-homologous molecule expressed on Th2 cells (CRTH2), and is known to cause contractions in smooth muscle in the airway *via* DP and to mediate the chemotaxis of eosinophils and basophils into the lung *via* CRTH2 (Matsuoka *et al.*, 2000 ▶; Hirai *et al.*, 2001 ▶). Haematopoietic PGD synthase (H-PGDS) is known to be expressed in mast cells, antigen-presenting cells and Th2 lymphocytes (Lewis *et al.*, 1982 ▶; Tanaka *et al.*, 2000 ▶) and produces PGD_2_ from cyclooxygenase-derived PGH_2_, a common precursor of various PGs (Kanaoka & Urade, 2003 ▶). Nonsteroidal anti-inflammatory drugs such as indomethacin and aspirin, both of which inhibit cyclooxygenase, suppress immune and inflammatory reactions by decreasing the production of all types of prostanoids, including cytoprotective and anti-inflammatory PGs, and induce adverse effects (Takeuchi *et al.*, 2001 ▶; Halter *et al.*, 2001 ▶). Therefore, H-PGDS-specific inhibitors are thought to be more useful lead compounds for anti-allergic and anti-inflammatory drugs, especially for the treatment of asthma, by decreasing the signals of PGD_2_ mediated by both DP and CRTH2. Aritake *et al.* (2006 ▶) reported the three-dimensional structure of human H-PGDS in complex with an orally active H-PGDS-specific inhibitor, 4-benzhydryloxy-1-[3-(1*H*-tetrazol-5-yl)-propyl]-piperidine (HQL-79), which acts as an anti-allergic drug, and elucidated the binding mode of HQL-79 within the catalytic pocket of H-PGDS. To develop more effective compounds, we have designed several novel H-PGDS inhibitors using HQL-79 as a lead compound and determined their binding modes in the enzyme–inhibitor complexes through high-resolution X-ray crystallographic analyses. During a series of high-quality protein crystallization experiments on the International Space Station (ISS) from 2003 to 2008 which were funded by the Japan Aerospace Exploration Agency (JAXA), we focused on high-resolution crystallization and obtained high-quality crystals of H-PGDS. In this report, we report the purification and microgravity crystallization and provide a summary of the preliminary X-ray diffraction analysis of complexes of H-­PGDS with three novel inhibitors for use in designing drugs based on its three-dimensional structure.

## 2. Materials and methods

### 2.1. Protein expression and purification

Human H-PGDS was expressed recombinantly in *Escherichia coli* and purified as described previously (Aritake *et al.*, 2006 ▶). In brief, *E. coli* BL21 (DE3) cells were transformed with the prepared plasmid carrying the cDNA of human H-PGDS. After the cells had been harvested, disrupted in phosphate-buffered saline by sonication and centrifuged, the resultant soluble fraction was applied onto a glutathione-Sepharose 4B column (GE Healthcare). H-PGDS bound to the resin was eluted with 50 m*M* Tris–HCl pH 9.0 containing 10 m*M* glutathione. As the preparation showed a broad band on native PAGE analysis, we further purified H-PGDS by Mono-Q HR5/5 chromatography (GE Healthcare) with a sodium chloride gradient from 0.1 to 0.2 *M* in 20 m*M* Tris–HCl at 293 K. H-PGDS eluted at around 0.15 *M* sodium chloride and separated into three peaks. The fractions from the first peak were used for crystallization. The final purified sample of H-PGDS showed a single band on SDS–PAGE under reducing conditions and native PAGE under nonreducing conditions. H-PGDS was concentrated to 3.0 mg ml^−1^ in 50 m*M* Tris–HCl pH 7.5 using a Centricon YM-10 membrane (10 000 nominal molecular-weight limit; Millipore) and stored at 277 K. The protein concentration was determined spectrophotometrically at 280 nm.

### 2.2. Inhibitors

HQL-79 was obtained from Cayman. Three H-PGDS inhibitors, compounds *A*, *B* and *C*, were synthesized at the Osaka Bioscience Institute. The formulae of the inhibitors are proprietary information. The IC_50_ values of the inhibitors were measured to be 400, 50 and 4400 n*M*, respectively, as described previously (Aritake *et al.*, 2006 ▶). The inhibitory effects of these inhibitors were stronger than that of HQL-79 (IC_50_ = 5900 n*M*; Aritake *et al.*, 2006 ▶).

### 2.3. Crystallization

H-PGDS was crystallized in a microgravity environment at 293 K inside a Thermal Electric Biological Universal incubator onboard the Russian Service Module on the ISS using JAXA’s microgravity crystallization experiments JAXA-NGCF#1 and JAXA-NGCF#2 for 12 weeks from January to April 2007 and for 11 weeks from August to October 2007, respectively.

**Figure 1:**
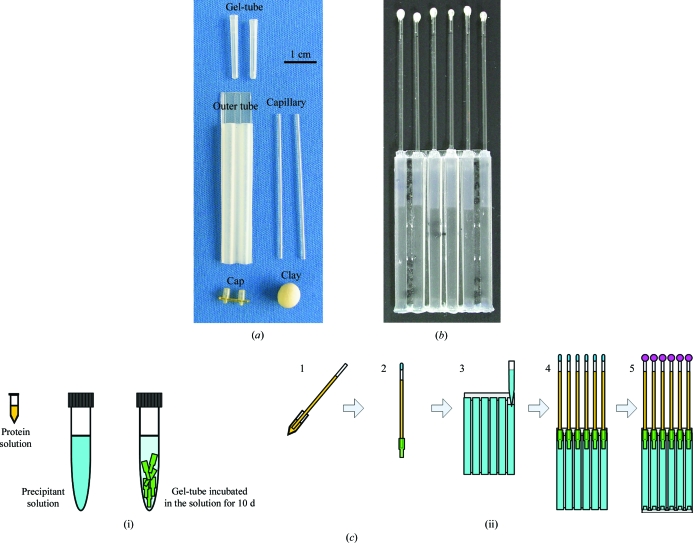
The setup and configuration of the counter-diffusion crystallization device, a JAXA crystallization box (JCB). (*a*) Parts of the crystallization device before assembly. (*b*) Outline of the assembled crystallization device for the microgravity experiment. It was mounted in an aluminium container, the Granada Crystallization Facility, and launched to the ISS. (*c*) Sample loading and crystallization-device setup. (i) Preparation of solutions. The protein solution (4.0 mg ml^−1^ human H-PGDS with or without 0.5 m*M* inhibitor in 150 m*M* sodium chloride, 15% PEG 6000, 5 m*M* dithiothreitol, 5 m*M* glutathione, 1% dioxane, 0.5 m*M* magnesium chloride and 20 m*M* Tris–HCl pH 8.0) and the precipitant solution (30% PEG 6000, 10 m*M* dithiothreitol, 10 m*M* glutathione, 1% dioxane and 1 m*M* magnesium chloride in 50 m*M* Tris–HCl pH 8.4) were prepared. The gel-tubes, which were polymerized agarose gels in a piece of plastic tubing, were incubated in 15% PEG 6000 solution containing 10 m*M* dithiothreitol, 10 m*M* glutathione, 2% dioxane, 1 m*M* magnesium chloride and 50 m*M* Tris–HCl pH 8.4 for 10 d before crystallization-device setup. (ii) Loading solutions and assembling the crystallization device. The protein solution was loaded into a capillary (1). The top of the capillary was tentatively sealed with clay and the gel-tube was plugged into the end of the capillary (2). The precipitant solution was loaded into the outer tube (3). The capillaries were inserted into the outer tube (4). The bottoms of the outer tubes were covered with caps and the top of the capillaries were completely sealed with epoxy adhesive (5).

For the JAXA microgravity experiments, we adopted the JAXA Crystallization Box (JCB) as a crystallization device (Tanaka *et al.*, 2004*a* ▶), which is a modification of the original capillary counter-diffusion method (García-Ruiz & Moreno, 1994 ▶; García-Ruiz, 2003 ▶; Ng *et al.*, 2003 ▶; Gonzalez-Ramirez *et al.*, 2008 ▶; Otálora *et al.*, 2009 ▶). The set-up configuration of the JCB is shown in Fig. 1 ▶. The crystallization condition was fixed to start after the samples had been placed in the microgravity environment (about 10 d after sample loading) by adjusting the precipitant concentration and the gel-tube length. A microseeding technique was applied as follows: small crystals of H-­PGDS were crushed in 5 µl twofold-diluted precipitant solution and diluted 100-fold with the precipitant solution; 1 µl of the microseed solution was then added to 11 µl protein solution. The number of capillaries for each H-PGDS inhibitor is shown in Table 1 ▶. The same crystallization condition was applied in the terrestrial control experiment.

### 2.4. Data collection

Diffraction data were collected at 100 K with an X-ray wavelength of 0.85 Å on the BL41XU beamline at SPring-8, Harima, Japan using an ADSC315 detector system. The crystals grown in the capillaries were extracted into artificial mother liquor (30% PEG 6000, 10 m*M* dithiothreitol, 10 m*M* glutathione, 1% dioxane and 1 m*M* magnesium chloride in 50 m*M* Tris–HCl pH 8.4). The concentration of PEG 6000 in the artificial mother liquor was calculated using a one-dimensional simulation program that estimates the time-course of the concentration change of the precipitant solution at a certain position in the capillary (Tanaka *et al.*, 2004*a* ▶). The method of harvesting crystals has been reported previously (Tanaka *et al.*, 2007 ▶). One crystal was selected with a nylon loop, briefly dipped into artificial mother liquor supplemented with 15% glycerol as a cryoprotectant for less than 10 s and plunged into a nitrogen-gas stream at 100 K. A total of 180 frames were collected using a crystal-to-detector distance of 150 mm with 1° oscillation. The diffraction images were integrated and scaled using the programs *DENZO* and *SCALEPACK* from the *HKL*-2000 suite (Otwinowski & Minor, 1997 ▶). We performed X-ray diffraction experiments on two or three crystals and summarize the best data for each complex in Table 1 ▶. Data sets were collected to the resolution range where *I*/σ(*I*) > 2 and *R*_merge_ < 50%.

## 3. Results and discussion

### 3.1. Visual inspection of crystals

**Figure 2:**
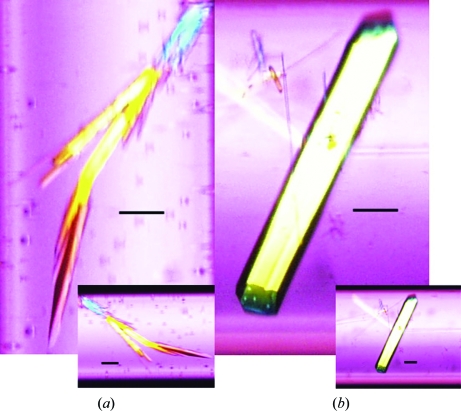
Crystals of H-PGDS grown under terrestrial (*a*) and microgravity (*b*) conditions. In the terrestrial experiment the size of the crystal was almost the same for all of the complexes. Crystals were first observed at around 10 mm from the bottom of the capillary after two weeks of crystallization setup and were observed at the top of the capillary (40 mm from the bottom) after 12 weeks of crystallization setup. The crystals had a tendency to form clusters. In the microgravity experiment plate-like crystals were grown with good morphology. The scale bars correspond to 100 µm.

Two or three crystals grew in every 10 mm of the capillaries both on terra firma and in microgravity. The dimensions of the crystals grown terrestrially and in microgravity were almost the same (0.3 × 0.1 × 0.05 mm). However, the crystals grown in microgravity did not form clusters (Fig. 2 ▶), suggesting that the crystal quality was improved by crystallization in microgravity. The number and size of the crystals did not change in the presence or the absence of H-PGDS inhibitors.

### 3.2. X-ray diffraction

The results of the X-ray diffraction are summarized in Table 1 ▶. All crystals chosen for diffraction data collection were of sufficient size and of good quality as judged by visual inspection. In the counter-diffusion method, crystals of better quality tend to grow at the opposite side to the gel-tube, in the section of the capillary where the concentration change of the precipitant is slower (Lopez-Jaramillo *et al.*, 2003 ▶). As a result, the crystals grown in capillaries at 30–40 mm from the gel-tube of the capillary were used for X-ray diffraction experiments in most of the cases. To avoid fluctuations in the data caused by crystal size differences, we chose microgravity-grown and terrestrial-grown crystals of the same size for X-ray diffraction experiments. Crystals of different space groups (*P*1) were obtained in the presence of inhibitors, owing to the binding of the inhibitor, compared with those grown in the absence of inhibitors (*P*2_1_).

Terrestrial-grown crystals of H-PGDS in the presence of inhibitor *B* and HQL-79 exhibited X-ray data sets to 1.8 and 1.5 Å resolution with mosaicities of 0.81 and 1.28, respectively (Table 1 ▶). Although the H-PGDS crystals grown in the absence of inhibitor (space group *P*2_1_) and in the presence of inhibitors *A* and *C* showed X-ray diffraction to 1.7, 2.0 and 2.0 Å resolution, respectively, we did not collect their X-­ray data sets because of relatively high mosaicity or poor-quality diffraction. In contrast, microgravity-grown crystals in the absence or the presence of inhibitors *A*, *B*, *C* and HQL-79 exhibited X-ray data sets to 1.5, 1.1, 1.1, 1.8 and 1.3 Å resolution with mosaicities of 0.54, 0.56, 0.62, 1.48 and 1.71, respectively (Table 1 ▶).

### 3.3. Affinity of inhibitors for H-PGDS

Among the microgravity-grown crystals, both the maximum resolution and the mosaicity of the X-ray diffraction data are relatively low in the complexes with inhibitors with low IC_50_ values (Table 1 ▶). This may be a consequence of immobilization of the catalytic pocket of H-­PGDS after binding the high-affinity inhibitors in the enzyme–inhibitor complexes, leading to the growth of well ordered crystals in microgravity.

Recently, novel inhibitors of H-PGDS have been generated by fragment-based drug design and are expected to contribute to novel drug development (Hohwy *et al.*, 2008 ▶). The best inhibitor showed an IC_50_ of 21 n*M* and is one of the most potent inhibitors described to date. Such high-affinity inhibitors may also improve the crystal quality of H-PGDS–inhibitor complexes in the microgravity environment, as shown in this report.

### 3.4. The counter-diffusion method in microgravity

The counter-diffusion method used in this report is based on the counter-diffusion of protein and precipitant along a capillary. Using this method in a convection-free environment such as microgravity or gels, uniform propagation of the supersaturation wave is expected in the capillary, which enables simultaneous screening for optimal conditions for protein crystallization. Moreover, in a convection-free environment a protein-depletion zone (PDZ) and an impurity-depletion zone (IDZ) are formed around the growing crystal, which are believed to be beneficial for improving crystal quality (McPherson, 1999 ▶; Chernov, 1998 ▶; Thomas *et al.*, 2000 ▶). In addition, these zones are formed much more significantly in a highly viscous solution such as PEG (Tanaka *et al.*, 2004*b* ▶) and with highly purified protein (Yamanaka *et al.*, 2009 ▶). Since the numbers of crystals that we have used here were very limited, we avoid any comparative statements; however, these might have positive effects on the growth of high-quality crystals in microgravity in our cases.

## 4. Conclusions

We obtained high-quality crystals of H-PGDS complexes with inhibitors by the counter-diffusion method in a microgravity environment. The counter-diffusion method in microgravity is the only way to obtain crystals of H-PGDS inhibitor complexes that diffract to 1.0–1.5 Å resolution. This will provide us with a better understanding of the binding mode of H-PGDS with inhibitors for future drug design. Structural refinement is currently in progress.

It is still difficult to identify any predictive technique to determine whether a crystallization experiment will benefit from microgravity; however, many examples of improved crystal quality in microgravity have been reported (Littke & John, 1986 ▶; DeLucas *et al.*, 1989 ▶; Day & McPherson, 1992 ▶; He & Carter, 1992 ▶; Borgstahl *et al.*, 2001 ▶; Kundrot *et al.*, 2001 ▶; Sauter *et al.*, 2001 ▶; Vergara *et al.*, 2003 ▶; Snell & Helliwell, 2005 ▶; Evrard *et al.*, 2007 ▶; Tanaka *et al.*, 2007 ▶; Meyer *et al.*, 2008 ▶). The complexes of human H-PGDS with high-affinity inhibitors are new examples of improved crystal quality in microgravity.

## Figures and Tables

**Table 1:** Summary of X-ray diffraction experiments on H-PGDS crystals The best data from two or three X-ray diffraction analyses of each H-PGDS–inhibitor complex are shown in the table. The data set was collected to the resolution range at which *I*/σ(*I*) >2 and *R*_merge_ < 50%.

							Unit-cell parameters							Maximum resolution (Å)	
Inhibitor	IC_50_ (n*M*)	Space or ground	No. of capillaries	Crystal dimensions (mm)	Position in the capillary† (mm)	Space group	*a* (Å)	*b* (Å)	*c* (Å)	α (°)	β (°)	γ (°)	Mosaicity (°)	Data set	Observed
Free	—	Space	5	0.3 × 0.03 × 0.03	20–30	*P*2_1_	48.5	47.3	184.3	90.0	97.0	90.0	0.54	1.5	1.5
		Ground	5	0.3 × 0.02 × 0.02	30–40	*P*2_1_	48.4	47.4	184.8	90.0	97.9	90.0	2.47	1.7	‡
*A*	400	Space	3	0.3 × 0.1 × 0.05	30–40	*P*1	47.0	48.6	89.6	96.2	90.0	90.0	0.56	1.1	1.1
		Ground	3	0.3 × 0.02 × 0.02	30–40	*P*1	48.8	47.2	93.4	87.1	80.1	90.1	3.39	2.0	‡
*B*	50	Space	3	0.1 × 0.1 × 0.05	30–40	*P*1	46.9	48.2	89.4	83.9	90.0	90.0	0.62	1.1	1.1
		Ground	3	0.1 × 0.1 × 0.05	30–40	*P*1	47.1	48.1	89.7	84.3	89.9	89.9	0.81	1.5	1.8
*C*	4400	Space	2	0.1 × 0.1 × 0.05	30–40	*P*1	46.9	48.0	88.9	84.3	89.9	89.9	1.48	1.4	1.8
		Ground	2	0.1 × 0.1 × 0.05	30–40	§	§	§	§	§	§	§	§	2.0	§
HQL-79	5900	Space	2	0.3 × 0.1 × 0.05	30–40	*P*1	46.9	48.7	89.3	95.8	89.9	90.1	1.71	1.3	1.3
		Ground	4	0.1 × 0.1 × 0.05	30–40	*P*1	47.0	48.3	89.2	95.7	90.2	89.9	1.28	1.5	1.5

† Position of the crystal from the bottom of the capillary.

‡ An X-ray diffraction data set was not collected because of high mosaicity.

§ An X-ray diffraction data set was not collected because of the poor quality of the diffraction.
